# Scalable nanolaminated SERS multiwell cell culture assay

**DOI:** 10.1038/s41378-020-0145-3

**Published:** 2020-06-01

**Authors:** Xiang Ren, Wonil Nam, Parham Ghassemi, Jeannine S. Strobl, Inyoung Kim, Wei Zhou, Masoud Agah

**Affiliations:** 1 Bradley Department of Electrical and Computer Engineering, Virginia Tech, Blacksburg, VA 24061 USA; 2 Department of Statistics, Virginia Tech, Blacksburg, VA 24061 USA

**Keywords:** Nanobiotechnology, Electrical and electronic engineering

## Abstract

This paper presents a new cell culture platform enabling label-free surface-enhanced Raman spectroscopy (SERS) analysis of biological samples. The platform integrates a multilayered metal-insulator-metal nanolaminated SERS substrate and polydimethylsiloxane (PDMS) multiwells for the simultaneous analysis of cultured cells. Multiple cell lines, including breast normal and cancer cells and prostate cancer cells, were used to validate the applicability of this unique platform. The cell lines were cultured in different wells. The Raman spectra of over 100 cells from each cell line were collected and analyzed after 12 h of introducing the cells to the assay. The unique Raman spectra of each cell line yielded biomarkers for identifying cancerous and normal cells. A kernel-based machine learning algorithm was used to extract the high-dimensional variables from the Raman spectra. Specifically, the nonnegative garrote on a kernel machine classifier is a hybrid approach with a mixed nonparametric model that considers the nonlinear relationships between the higher-dimension variables. The breast cancer cell lines and normal breast epithelial cells were distinguished with an accuracy close to 90%. The prediction rate between breast cancer cells and prostate cancer cells reached 94%. Four blind test groups were used to evaluate the prediction power of the SERS spectra. The peak intensities at the selected Raman shifts of the testing groups were selected and compared with the training groups used in the machine learning algorithm. The blind testing groups were correctly predicted 100% of the time, demonstrating the applicability of the multiwell SERS array for analyzing cell populations for cancer research.

## Sensors: improved Raman spectroscopy for cell culture analysis

Researchers in the USA have developed a cell culture platform which enhances spectroscopic analysis, making it possible to carry out molecular profiling of biological samples without any labeling. A team led by Masoud Agah and Wei Zhou of Virginia Tech incorporated nanolaminated plasmonic structures into the surface of a multi-well cell culture plate. This modification improves the performance of Raman spectroscopy, facilitating more sensitive and precise biochemical profiling, and the resulting spectra were analyzed via machine learning. The team tested their approach by using it to discriminate samples of normal breast tissue, breast cancer cells, and prostate cancer cells. The analysis distinguished the cell types with over 90% accuracy. This approach can be used for label-free analysis of diagnostic biomarkers to identify specific cancer subtypes, and further work may make it possible to analyze samples of mixed cells.

## Introduction

Cancer is one of the leading causes of death worldwide and is involved in approximately 9 million deaths each year^1^. Current therapies for cancer are rarely curative and suffer from limitations such as the lack of specific drug targets, off-target nonspecific drug toxicities, and multidrug resistance, all of which can benefit from a better analysis and understanding of tumor populations^2^. Distinguishing between cancerous cells and normal cells at an early stage of diagnosis is critical for early prediction, early intervention, and ultimately the suppression of the proliferation and metastatic potential of primary tumors in potential cancer patients. Based on a decent diagnosis of cancerous cells in biopsy samples, specific treatment plans can be determined for each cancer patient, which is essential in “precision medicine”^3^. The characteristics of different subtypes of tumor cells can facilitate the accurate diagnosis and effective treatment of specific tumors. The precise diagnosis of cancer cells from different stages or subtypes is a challenging task in clinical trials. To diagnose cancer cells from normal cells, the cell culture assay has been widely used in clinical practice with label-based immunohistochemistry (IHC) studies, which target specific cell protein expressions. However, the diagnosis of different cancer subtypes, such as different subtypes of breast cancer or prostate cancer, requires further labeling techniques^4^. Labeling techniques for both breast cancer and prostate cancer cells are widely used in clinical practice.

Multiwell arrays for cell cultures, either as commercially available products or custom-made polydimethylsiloxane (PDMS) devices, have played a significant role in basic cancer research and clinical cancer diagnosis. Multiwell arrays allow cell growth, metastasis, migration, and drug testing of a large number of variables in parallel^5^. In addition, multiwell arrays require only 50–60 µL samples per well, thus conserving biopsy tissue and analysis cost. There have been efforts to integrate different sensing mechanisms with multiwell cell culture substrates to perform label-free analysis. Some of these sensing techniques include bioimpedance spectroscopy, piezoelectric sensors, and optical biosensors. For example, microelectrode arrays (MEAs) have become commercially available for bioimpedance measurements of cultured cells. Multiwell platforms with embedded MEA can be used to monitor the long-term bioimpedance variation during cell metabolism and proliferation and can also be used to study the drug treatment response^5^. Surface coating of MEAs with nanoparticles can facilitate studies of cellular interactions with the extracellular matrix (ECM) via bioimpedance spectroscopy. Piezoelectric biosensors can also be integrated into cell culture platforms for cell subtype recognition. Usually, a piezoelectric crystal is functionalized to capture a certain type of cell. Researchers found that the cells on a quartz crystal will generate a unique oscillating frequency, which can be used to identify a type of cell with common surface markers^6^. However, the functionality of the piezoelectric crystal relies on the selection of the surface markers of cells. In addition, the sensitivity of the crystal itself determines the sensitivity of this mass-based transducer for sensing cultured cells^7^. Another sensing technique utilizes optical sensors to detect cancer cells, such as photonic crystal biosensors^8^. The optical biosensor can also be made onto the multiwell culturing plate^9^. After the cells attach to the sensors, the proliferation of the cancer cells can be observed by proper imaging methods^9^. In this paper, for the first time, we report the integration of scalable surface-enhanced Raman spectroscopy (SERS) substrates with multiwell cell culture platforms for the label-free SERS profiling and machine learning classification of subtypes of living cancer cells.

By the plasmonic enhancement of both the excitation and inelastic Raman scattering processes of molecules in hot spots of metal nanostructures, SERS can increase the sensitivity of Raman spectroscopy by many orders of magnitude with a detection limit down to the single-molecule level^10–12^. Therefore, SERS has emerged as an ultrasensitive molecular spectroscopy technique for the label-free monitoring and analysis of living biological systems, which can potentially mitigate the limitations of conventional fluorescence techniques^13,14^. H. Wu et al. developed a gold SERS substrate on graphene nanosheets that showed sufficient sensitivity to differentiate between breast cancer cells and breast cancer stem cells^15^. D. Gracias et al. developed a mechanical trap SERS array on a quartz substrate to collect Raman scattering of the trapped breast cancer cell line MDA-MB-231^16^. C. Zhong et al. used magnetic focusing by magnetic beads coated with cancer cell biomarkers to trap cancer cells and collected SERS spectra of the trapped cells^17^. Unfortunately, trapping cells by means of a mechanical trap or antibody affiliated capturing method adds additional noise to the Raman spectra, which reduces the consistency of the Raman detection or imaging of the cancer cells. W. Zhou et al. developed scalable high-performance SERS substrates based on multistack vertically oriented nanogap hot spots in metal-insulator-metal (MIM) nanolaminated plasmonic structures^18,19^. The recently published work reports the novelty in the physics of refractive-index-insensitive nanolaminated SERS substrates targeting the label-free spatiotemporal Raman biochemical analysis of living biological systems^19^. Nanolaminated SERS substrates based on multiresonant multilayered metal-insulator-metal plasmonic nanostructures can support optically dense and uniform arrays of hot spots with very large and consistent SERS enhancement factors (>10^7^) that are insensitive to a wide range of background refractive indices (*n* = 1.30–1.60). Both the numerical simulations by 3D finite-difference-time-domain (FDTD) and experimental results indicated that the nanolaminated SERS substrate has high sensitivity and uniformity simultaneously. Both high sensitivity and good uniformity of SERS hot spots were achieved by optically dense and highly uniform arrays of vertically oriented hot spots. SERS can distinguish the differences in biochemical environments between the cytosol, nucleus, and extracellular matrix through molecular vibrational signatures^13^. Molecular event dynamics of apoptosis in living cancer cells, including protein denaturation, proteolysis, and DNA fragmentation, can be observed in real-time.

The analysis of SERS spectra is usually performed by principal component analysis (PCA). PCA is a popular unsupervised learning approach. PCA has been used as a dimension-reduction tool that can reduce a large set of variables to a small set that contains most of the information in the large set. Since the Raman peaks at different wavenumbers can be viewed as high-dimensional variables, machine learning algorithms have been applied to analyze the data from SERS spectra. J. Liu et al. published studies on cancer research using SERS nanoparticles for Raman imaging and predicted that machine learning methods can accurately identify tumor SERS imaging, which represents unique biomarker expression signatures at the molecular level^20^. N. Othman et al. used the k-nearest neighbor (k-NN) algorithm to analyze SERS spectra integrated with PCA^21^. However, the k-NN algorithm is nonparametric without a particular model to fit.

In this report, we demonstrated a novel nanolaminated SERS substrate integrated multiwell cell culture assay for label-free Raman profiling and classification of living cancer cells. A nonnegative garrote on the kernel machine (NGK) algorithm was used to analyze the SERS spectral data collected from two triple-negative breast cancer (TNBC) cell lines, i.e., one normal breast epithelial cell line and one prostate cancer cell line.

## Results

### Cell lines

**Fig. 1:**
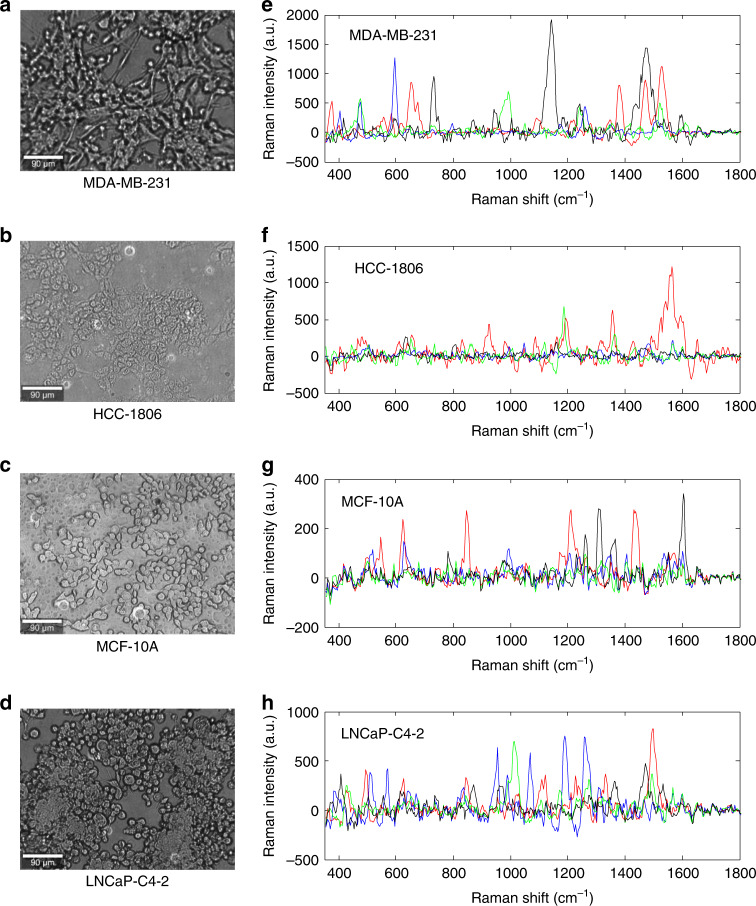
Bright field images of the cells and the Raman measurement results. Morphology images of the cells in multiple wells: **a** MDA-MB-231, **b** HCC-1806, **c** MCF-10A, and **d** LNCaP-C4-2 cells and four examples of the Raman measurement results of each cell line: **e** MDA-MB-231, **f** HCC-1806, **g** MCF-10A, and **h** LNCaP-C4-2 cells

We used a modified version of a PDMS multiwell array incorporated with nanolaminated plasmonic structures to measure the Raman spectra of cultured cells. The bright field images of the cell morphologies shown in Fig. 1a-d were taken through an objective lens (×10) before the Raman measurements. Due to the different growth rates of each cell line, the final cell density may vary even though the seeded cell suspension solutions were approximately 4 × 10^5^ cells/mL. The representative spectra of each cell line are shown in Fig. 1e-h. Each color represents an independent Raman spectrum. The Raman spectrum shown here is the data after background subtraction. The Raman spectrum of the SERS substrate without cells is recorded as the background (Fig. S1 in the supplementary information). The remaining spectrum is the effective information of the Raman spectrum of the cells and Rayleigh peak. The data selected between 400 and 1800 cm^−1^ represent the Raman spectrum of the cells. Different spectra for each cell line show different Raman peak positions and intensities. The single and/or combination of peaks act as a fingerprint to identify a cell. The Raman peaks can be correlated to possible biochemical attributes, as listed in Table 1^22–36^. Interestingly, more than 3 different features are observed in each case. This finding is highly associated with the larger sizes of cells compared to those of nanoantennas so that nanoantennas can collect signals from different nanoscale regions of the cells where the distributions of biomolecules may be different.

**Table 1:** Selected Raman peaks and the possible correlated biochemical attributes

Peaks (cm^−1^)	Possible attribution	References
588	Acetoacetate	^22^
679	Proteins	^23,24^
699	Phosphatidylcholine, lipids	^25^
757	Cytochrome, tryptophan	^26,27^
766	Fumarate	^22^
878	C–C–N^+^ stretching	^27^
889	Glycine	^22,29,30^
1020	Phenylalanine	^28–31^
1080	Proteins: stretching C–N; carbohydrates: stretching C–O	^27,32,33^
1158	Acetoacetate	^22^
1230	Amide III (β-sheet)	^28,34^
1260	Fumarate	^22^
1380	Proteins: twisting (CH_2_, CH_3_)	^33^
1460	Histidine	^28^
1670	Amide I: C=C tyrosine, tryptophan, lipids, stretching (C=C) olefinic	^27,32,36^
1740	Lipids, C=O ester	^27^

### Data analysis

**Fig. 2:**
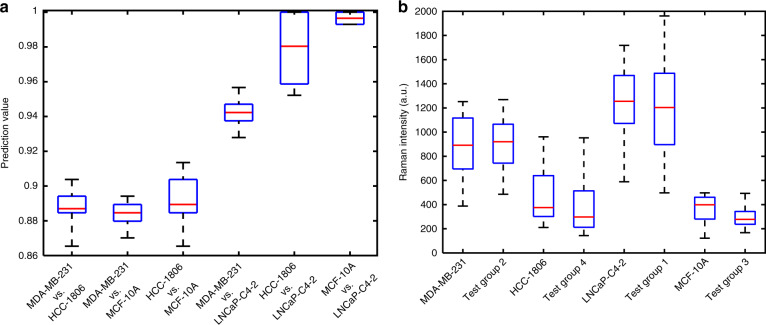
The comparison results of every two sets of cell lines. **a** The prediction values between different cell types. The box lines from bottom to top represent the percentiles: minima, Q25, Q50 (median, red lines), Q75, and maxima. **b** Blind testing groups 1–4 compared to the original experimental groups. Each blind testing group was matched to one of the cell lines

The NGK machine learning method was used to analyze the SERS spectra of the four cell lines. The comparison and predictions were performed between every two sets of cell lines. The Raman spectra with peak intensities (a.u.) over 2000 will be identified as outliers. The prediction values in Fig. 2a include the outliers in each sample, which already reach over 88%. The prediction values without outliers (data not shown in Fig. 2) reached 91% (MDA-MB-231 vs. HCC-1806), 96% (MDA-MB-231 vs. MCF-10A), and 96% (HCC-1806 vs. MCF-10A). Defining the outliers in Raman spectroscopy is based on experience and SERS substrate performance.

The box plot in Fig. 2a depicts the prediction values of the two cell lines. A comparison of two TNBC cell lines MDA-MB-231 and HCC-1806 indicates a prediction value of 86.5–90.4% in the 10-fold cross validation (CV). The NGK machine learning 10-fold CV results showed a prediction value of 87.0–89.4% between MDA-MB-231 and MCF-10A and a prediction value of 86.5–91.4% between HCC-1806 and MCF-10A. The prediction value between the breast cancer cell line MDA-MB-231 and the prostate cancer cell line LNCaP-C4–2 reached 92.8–95.7%. HCC-1806 and LNCaP-C4-2 showed higher prediction values of 95.2–100%. MCF-10A and LNCaP-C4-2 showed the highest prediction value of 99.3–100% among all the comparisons. The prediction values between breast cells and prostate cancer cells (the three boxes on the right side of Fig. 2a) are higher than those of different breast cells (the three boxes on the left side of Fig. 2a). The highest difference falls into the prediction of prostate cancer and normal breast cells (MCF-10A vs. LNCaP-C4-2 in Fig. 2a).

The purpose of the blind test is to determine whether our presented technique can correctly identify the cell lines. Blind testing groups with the four cell lines were used to validate the reproducibility and reliability of the biostatic SERS analysis of the cells. After collecting the data from newly cultured cells, the Raman intensities at the specific peaks in Table 1 were calculated and compared with the original measurement results. Notably, although the peak location of an isolated functional group is typically known, the actual peak locations may slightly differ due to interactions and bonding with its neighbors. The peaks located at a specific wavenumber can have a shift of 3–5 cm^−1^ due to the molecular interaction with the SERS substrate. Therefore, to increase the robustness of the measurement results, the peaks were selected by comparing the intensities of the Raman shifts with the two wavenumbers nearby. Then, the highest intensity number of the three Raman shifts was identified as the intensity. The box plots in Fig. 2b demonstrate the minima (lower black line), Q25 (lower blue line), Q50 (median, red lines), Q75 (upper blue line), and maxima (upper black line) values of the experimental and testing groups.

The box plot in Fig. 2b depicts the paired blind test groups (TGs: TG1, TG2, TG3, and TG4) with the standard measurement groups. By matching the TGs with the measured cell lines, TG1 was recognized as LNCaP-C4-2 (*t* = 1.99, *p* = 0.024); TG2 was recognized as MDA-MB-231 (*t* = 1.22, *p* = 0.099); TG3 was recognized as MCF-10A (*t* = 5.79, *p* < 0.0001); and TG4 was recognized as HCC-1806 (*t* = 1.48, *p* = 0.071). This result is a 100% match with the record before the TG experiments.

## Discussion

For the practical use of SERS substrates for biological and clinical applications, high sensitivity and uniformity along with biocompatibility and a high-throughput screening ability are required^12^. Nanolaminated plasmonic structures can simultaneously achieve the two major requirements of SERS performance, that is, sensitivity and uniformity by out-of-plane engineered multiple vertically oriented nanogap hot spots based on Au-SiO_2_-Au building blocks^19^. Our recently published work in Nano Letters reports a novelty in the physics of RI-insensitive nanolaminated SERS substrates targeting the label-free spatiotemporal Raman biochemical analysis of living biological systems^19^. The majority of SERS substrates are too small to be incorporated. However, our nanolaminated SERS substrate can effectively solve this problem. A cost-effective, facile, and time-saving fabrication process is available for clinical studies, and this lithography-free method on a large scale enables the incorporation of SERS into a PDMS multiwell array for parallel testing. The combination of molecular information from different regions can uniquely represent a certain type or status of cells. Out-of-plane engineered nanostructures interfacing with cells show better adhesion to cell membranes than flat surfaces^37,38^. This property provides lower cell mobility on the device and accordingly forms a microenvironment underneath the cells where biomolecules can be efficiently sensed. The contact between the SERS substrate and the cell membrane and the transmembrane proteins generates an interaction between the chemicals and nanopillars, which can effectively create unique biosignatures in the SERS spectra. The peaks appearing at different Raman shifts are correlated with the cell lipid membrane components and specific molecules, including proteins and specific DNA or RNA sequences^39^. The SERS detection of cancer-specific metabolic profiles involving glucose, glutamine, asparagine, aspartate, acetate, and lactate, for example, are likely to contribute to the SERS biosignatures. The preferential glycolytic activity of tumor cells, first described as the Warburg effect, means that the primary energy source for tumor cells is glucose^40^. Tumor cells take up glucose at higher rates than normal cells and secrete higher levels of lactate, resulting in the localized acidification of their microenvironment. Tumor cells in culture shunt glutamine through these pathways and satisfy their increased metabolic demands for nitrogen via the high uptake of glutamine and asparagine. Tumor cells maintain high levels of de novo fatty acid synthesis and histone acetylation, which are sustained by elevating the uptake of acetate from the culture medium. The Raman wavenumbers for these specific proteins are correlated to their specific amino acids. Based on the NGK method selected Raman wavenumbers, the peaks from proteins and amino acids are found at wavenumbers of 588, 679, 889, 1020, 1080, 1158, 1380, and 1460 cm^−1^. The genetic differences among the four cell lines also contribute to the different expression of transmembrane proteins. MDA-MB-231 cells express P-cadherin and/or cadherin-11 (without N-cadherin), which promotes motility and invasiveness as an aggressive TNBC cancer cell line^41^. At the same time, MDA-MB-231 cells express vimentin (Basal B), while HCC-1806 is a special non-Basal A, non-Basal B epithelial TNBC cell line without vimentin expression^42^. MCF-10A is derived from the mastectomy of a benign tissue, representing normal epithelial breast cells, which is also Basal B in molecular classification. MCF-10A cells demonstrate spontaneous morphological changes in response to different confluence statuses. The analysis of Raman spectra indicates good separation between the breast cancer and breast normal cells. In addition, the difference between the Basal B and non-Basal B subtypes can also be differentiated by Raman scattering. The detection sensitivity of Raman spectroscopy and the machine learning algorithm is sufficient to identify the different subtypes of breast cancer cell lines. Prostate-specific membrane antigen (PSMA) is a protein encoded by the float hydrolase-1 gene that exists in the LNCaP-C4-2 cell line^43^. The genetic information carried by different combinations of adenine, thymine, guanine, and cytosine is represented by Raman wavenumbers of 1423, 1210, 1315, and 1533 cm^−1^, respectively. Our Raman wavenumbers with possible correlated biochemicals listed in Table 1 match the possible biochemical attributes from multiple studies in the literature^22–36^. The high prediction values and accurate prediction of blind testing groups can be explained by the biological properties of the cells. As illustrated in the SEM image in Fig. 4, the membrane proteins, including cadherins, reach the multilayered MIM nanopillars. The filopodia and lamellipodia are extended to cover the nanopillars, which enables the chemical exchanges sensed by Raman spectroscopy. The culture medium under filopodia and lamellipodia is enriched with cell-secreted metabolites and cell signaling mediators, including proteins, small molecules, and exosomes. Therefore, the multilayered MIM nanopillars can sense the exchange of cellular products across the plasma membrane into the culture medium and represent the biosignatures of the specific cells by the Raman signals.

Raman spectra can be viewed as high-dimensional variables with dependent relationships. Applying machine learning techniques to Raman spectroscopy data analysis can achieve results with predictive power. A machine learning method that has been used in SERS data analysis is the support vector machine (SVM) algorithm. S. Li et al. presented a genetic algorithm analyzing the histogram of the peaks in the Raman spectra of bladder cancer^44^. However, finding the separating hyperplane of the SVM method for training the data sets is very time-consuming. The performance of SVM depends on tuning parameters. Another popular supervised learning classifier is a neural network classifier (NNT). NNT also shows promising applications for molecular level biosensing. However, neural networks extend the basic idea used in the perceptron model, in which the input is directly connected to the output. NNT comprises multiple layers of logistic regression models with continuous nonlinearities. Therefore, the performance of NNT depends on the number of layers, the estimates of the weight vector, and the active functions. Both SVM and NNT methods assume the independence of outputs, while our NGK approach does not. Hence, SVM and NNT cannot be applicable when there exists a possible dependence among outputs. In addition, both SVM and NNT are discriminative classifiers but are not dimension-reduction tools. NGK can be considered a hybrid approach that is a mixed nonparametric model and a kernel machine tool^45^. Using a training set, we built NGK classifiers that were required to estimate a nonparametric function but that did not assume a particular function form. This nonparametric function is estimated via a Gaussian process, which is known as a family of nonparametric functions.

The real-time characterization and differentiation of biochemical properties of living cells remain a significant challenge in biological science and technology. In particular, monitoring the biochemical signatures and behaviors of cells will help the identification of upregulated or downregulated pathways, the discovery of biomarkers related to specific pathologies or diseases, or the understanding of cellular responses to environmental changes. Such studies will facilitate the investigation of the underlying mechanisms behind different diseases and eventually contribute to early medical diagnostics. In situ detection of the biochemical properties of cells at subcellular resolution imposes difficult constraints on the measurement tools. However, the multiwell array integrated with nanolaminated SERS substrates presented in this paper can offer a label-free and potentially automated approach to the high content analysis of individual patient tumors and a new approach for the rapid identification of diagnostic biomarkers. In addition, this technique can be suitable for future development to evaluate anticancer drug sensitivity in tumor cell populations.

The multilayered MIM nanolaminated SERS substrate incorporated with a PDMS multiwell array acquired stable Raman spectra of prostate cancer, breast cancer, and normal breast epithelial cells. The introduced kernel-based machine learning technique successfully extracted the significant peaks from the Raman spectra to identify the cell types. The results of blind test groups proved the stability, reliability, and reproducibility of our technique. The multiwell array can be useful in analyzing biopsy samples with small amounts of samples. In addition, this device can be used to identify specific cancer subtypes. Analyzing cell mixtures that are present in a tissue sample by SERS is the next phase of development. Previously, we performed the biomechanical analysis of cell mixtures of normal and cancerous breast tissue obtained from patients with breast cancer^45^. We demonstrated that the biophysical attributes can be used to differentiate between the tumor tissue and adjacent normal tissue with machine learning algorithms. We envision that multiwell SERS technology will provide molecular profiling biostatistical information within spatially resolved SERS spectra to predict the presence of tumor cells in a heterogeneous cell population. In the future, applying SERS to biopsy samples might help define the margins of the tumor tissue, an important clinical application. However, the introduction of SERS bioanalysis technology into intraoperative real-time diagnosis is still very challenging, requiring (1) the advanced engineering of flexible SERS device arrays to seamlessly interface with tissues, (2) significant instrumentation engineering for the real-time Raman spectroscopy mapping of tissues integrated with SERS device arrays, and (3) efficient and adaptive machine learning techniques to allow for real-time closed-loop bioanalysis and diagnostics. We believe the results demonstrated in this work provide some promising possibilities for clinical applications.

## Materials and methods

### Cell culture

The breast cancer cell line MDA-MB-231 (passage #8, American Type Culture Collection (ATCC, Manassas, VA), provided by Dr. Yasmine Kanaan, Howard University College of Medicine) was grown in F12:DMEM (Lonza, Basel, Switzerland) with 10% fetal bovine serum (FBS), 4 mM glutamine and penicillin-streptomycin (100 units per mL). The African American breast cancer cell line HCC-1806 (passage #5, ATCC, provided by Dr. Yasmine Kanaan, Howard University College of Medicine, Washington, DC) was grown in ATCC-formulated RPMI-1640 medium with 10% FBS. Both MDA-MB-231 and HCC-1806 are estrogen receptor-negative (ER-), progesterone receptor-negative (PR-), and human epidermal growth factor receptor 2-negative (HER2-) breast cancer, named triple-negative breast cancer (TNBC). MCF-10A cells (passage #20, Lombardi Comprehensive Cancer Center, Georgetown University) were grown in F12:DMEM with penicillin-streptomycin (100 units per mL), 2.5 mM L-glutamine, 20 ng/mL epidermal growth factor (EGF), 0.1 μg/mL cholera toxin, 10 μg/mL insulin, 0.5 μg/mL hydrocortisone, and 5% horse serum. The prostate cancer cell line LNCaP-C4-2 (passage #11, provided by Dr. Bethany Kerr, Wake Forest University School of Medicine, Winston-Salem, NC), green fluorescence protein (GFP) by lentiviral transduction, was grown in RPMI-1640 (L-glutamine) with 10% FBS and 1% PenStrep (100 U/mL penicillium and 100 μg/mL streptomycin). All of the cells were grown in T-25 cm^2^ culture flasks at 37 °C in a 5% CO_2_ in air atmosphere until the cells were ready for subculture. The morphology of the cells was observed before trypsinization. The cells were then detached from the flask with a trypsin-EDTA solution (Sigma Aldrich, St. Louis, MO). MDA-MB-231, HCC-1806, MCF-10A, and LNCaP-C4-2 cells were trypsinized at 37 °C for 2 min, 8 min, 15 min, and 5 min, respectively. The passage numbers used for the 4 cell lines in this manuscript were below 20. The variation in the properties of cell lines can still be considered stable^46,47^. The culture environment was kept constant during the cell cultures. Furthermore, the experimental environment was also kept identical during all these SERS experiments. Because the cells are living cells, they actively divided during the experiments.

### SERS substrate fabrication

**Fig. 3:**
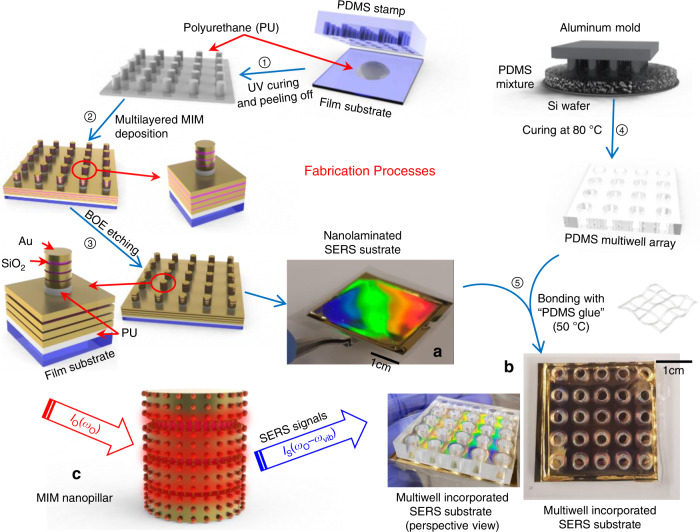
SERS substrate and multiwell array fabrication and assembly processes

The entire fabrication process was recently published^18^. Three-dimensional finite-difference time-domain (FDTD) analysis and experiments were performed to optimize the dimensions of the multilayered MIM nanolaminated plasmonic structures^48^. The fabrication procedure is briefly described in Fig. 3 (the detailed fabrication procedures are available in the supplementary information). Step ➀: UV-curable polyurethane (PU) was squeezed onto a flexible and optically transparent polyester (PE) film with a thickness of 100 µm and then was molded using a PDMS stamp to make nanopillar arrays (NPAs). The sample was cured by UV for 10 min, and the PDMS stamp was then peeled off. An additional heat-curing process was performed in a convection oven at 80 °C overnight. Step ➁ : we deposited alternating layers of Au and SiO_2_ by electron-beam deposition on the NPA. The nominal thicknesses of the four Au layers and the three SiO_2_ layers were 30 nm and 6, 8, and 12 nm from bottom to top. Step ➂: A buffered oxide etchant (BOE) solution was used to etch the SiO_2_ layers for 30 s. This process activates latent hot spots buried in the nanogaps, which are physically unapproachable for molecules before etching. The photo image (a) in Fig. 3 highlights a representative sample size fabricated on a flexible and optically transparent substrate. The vivid rainbow color diffraction pattern reflects the uniformity of periodic nanostructures on the sample over a wafer-scale area. As shown in Fig. 3c, nanolaminated plasmonic structures can strongly concentrate local optical fields in the nanogaps to generate plasmon-enhanced Stokes Raman scattering signals I_S_(ω_O_−ω_vib_) of the analyte molecules from the excitation light I_O_(ω_O_), where ω_O_ and ω_vib_ are the excitation laser frequency and molecular vibration frequency, respectively. The vertical stacking of MIM building blocks and activation of nanogaps form multiple vertically oriented nanogap hot spots. This unique geometrical configuration enables our SERS substrate to achieve both sensitivity and uniformity and reduce the footprint and volume of the device, thereby allowing signal acquisition from the different nanoscale environments of cells.

### Multiwell array fabrication

The multiwells were molded by a PDMS molding replica with an aluminum mold. The aluminum mold has 4 by 4 cylindrical pillars with a diameter of 4 mm. The PDMS prepolymer (SYLGARD® 184 silicone elastomer, Dow Corning, Midland, MI) and curing agent (SYLGARD® 184 silicone elastomer curing agent, Dow Corning, Midland, MI) were mixed at a weight ratio of 10:1. Tridecafluoro-1,1,2,2-tetrahydrooctyl-1-trichlorosilane (TFOCS, Fisher Scientific, Hampton, NH) was coated on the surface of the silicon wafer (single-side polished 4-inch silicon prime wafer) and the aluminum mold for the easy release of PDMS. The PDMS mixture was then placed in a vacuum container for 30 min to remove all the air bubbles. The degassed PDMS mixture was poured onto the silicon wafer with aluminum molds placed on the wafer surface and placed on a 90 °C hotplate for 12 h for the solidification of PDMS. Then, the PDMS was peeled off from the silicon wafer and the aluminum mold (Fig. 3 step ➃). Each PDMS well was 4 mm in diameter and 4 mm in height. The PDMS multiwell was then trimmed to match the dimensions of the SERS substrate.

### Multiwell cell culture platform integration

We employed a new fabrication method to prepare a PDMS multiwell array-incorporated SERS substrate. Notably, we used a thin PDMS layer as an intermediate adhesive film to bond the PDMS multiwell array and the SERS substrate coated with gold. The PDMS prepolymer and curing agent were fully mixed and preserved in a refrigerator (−18 °C), which guaranteed full mixing. The freshly made PDMS mixture can be cured at 60 °C in 2 h; however, the premixed PDMS preserved in the refrigerator can be cured at 60 °C within less than 30 min, which can function as the “PDMS glue” (Fig. 3 step ➄)^49^. The secondary curing temperature was kept below 70 °C to protect the nanostructures of the SERS substrate. The preserved PDMS mixture was injected from a syringe with a 0.3 mm needle tip to dispense a thin line on the PDMS multiwell array. Then, the assembled device was transferred to a 60 °C hot plate with a 200 g weight on the top to improve contact. After 30 min of curing, the assembled device was ready for cell culturing. The photo image (b) in Fig. 3 shows a final SERS substrate successfully incorporated with a PDMS multiwell array, and the dark feature of the sample in the visible range confirms broadband light absorption.

### Experimental setup

For the SERS measurement of different cell lines, each cell line was harvested and cultured on the multiwell array incorporated with the SERS substrate. The cells were harvested and diluted to a concentration of ~4 × 10^5^ cells/mL. Each well contained a volume of 60 µL. The device was then placed in an incubator at 37 °C with 5% CO_2_ in an air atmosphere for 12 h to allow the cells to attach to the nanoantennas. After attachment, the device was mounted on a piezo-driven scan stage for the SERS measurement. Cell mitosis was observed within 30 min under a bright field microscope, which confirms the cell viability on the SERS substrate. A ×10 objective was used, and the power of the infrared laser (wavelength: 785 nm) was 2.5 mW. The scan stage was manually adjusted to search for the Raman signals. The acquisition of a single spectrum from the points of interest was performed by taking 10 accumulations of 2 s integration time.

**Fig. 4:**
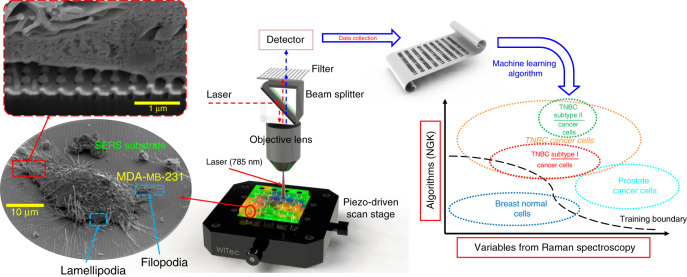
The experimental setup for cancer cell analysis by multiwell array-incorporated SERS substrate with a kernel-based machine learning algorithm

SEM characterization was performed using either an LEO (Zeiss) 1550 field emission scanning electron microscope (FESEM) or a Helios 600 Nanolab dual-beam (FEI) with an in-lens detector (represented in Fig. 4). The cultured cells were rinsed with warm (37 °C) PBS solution twice before fixation. The cells were chemically fixed with 2.5% glutaraldehyde in PBS solution for 1 h at room temperature. The samples were rinsed with PBS twice and postfixed with 1% osmium tetroxide (OsO_4_), followed by dehydration in a graded ethanol series from 15 to 100% (each condition was carried out for 15 min). After dehydration, the samples were dried by a critical point drier (CPD) in liquid CO_2_. PtPd (5 nm) was sputtered, and the samples were directly mounted on specimen holders. The enlarged SEM image in Fig. 4 shows that the lamellipodia and filopodia are extended to the nanoantenna array. The surface proteins are stretched and attached to the top of the nanoantennas. The cross section of the SEM image shows an interface between the cell and the SERS substrate. Some part of the membrane is attached to the top of the nanoantennas, while some part of the cell membrane is suspended above the nanoantennas. This suspension space will be filled with cell culture medium. Therefore, cell metabolism and mitosis will keep exchanging nutrition across the cell membrane.

### Data analysis

As demonstrated in Fig. 4, we use the NGK machine learning algorithm to analyze the Raman spectral data. NGK nonparametrically models unknown interaction terms among high-dimensional variables. To achieve the biostatic analysis of high-dimensional SERS signals of cells, we developed an NGK classifier by connecting a kernel machine with the multivariate nonparametric regression model^50^. This kernel-based approach can automatically model unknown and complicated interactions, which provides flexibility for both parametric and nonparametric models. Furthermore, the flexibility also applies to additive and nonadditive nonparametric models. If there are no complicated interactions or nonparametric models, then this kernel-based model automatically becomes an additive model or parametric model.

Consider *n* intensities for each cell type *t*, *t* = 1, …,*T*, and *p* represents the wave/peak variables data set (*y,X*_*t*_), where *X*_*t*_ = [*x*_1*t*_*, x*_2*t*,_ *…, x*_*pt*_]_,_ *x*_*jt*_ = [*x*_*j*1*t*_*, x*_*j*2*t*_*, …, x*_*jnt*_]^T^ is a n×1 vector for the *j*th variable, and *j* = 1, …,*p*. In our study, the range of the Raman shifts of interest was *n* = 104, *T* = 5, and *p* = 842. According to the representer theorem, the nonparametric regression model can be expressed as

$$\Pr \left( {y = t{\mathrm{|}}X_t} \right) = H\left[ {f\left( {X_t} \right)} \right] = H\left( {K\alpha } \right)$$

where *f* (*X*_*t*_) is the unknown nonparametric function, *K* is the kernel matrix corresponding to the function Hilbert space, and *α* is an unknown parameter. Our kernel can be expressed in a nonlinear function form because the Gram matrix

$$K\left( {\xi ,X_t} \right) = g\left( {\mathop {\sum}\limits_{j = 1}^p {\xi _jD^j} } \right)$$

where *g* is a known function (i.e., Gaussian form) and *D*^*j*^ is the matrix with the (*k,l*)th entry

$${d}_{{\mathrm{kl}}}^{\mathrm{j}} = - \left( {x_{\mathrm{jk}} - x_{\mathrm{jl}}} \right)^2$$

The objective function for NGK is to estimate *f* (*X*_*t*_), which is in the Hilbert space, for minimizing the objective function,

$${\mathrm{log}}\left( {\mathop {\prod}\limits_{t = 1}^T {{\mathrm{H}}\left[ {K\alpha \left( \xi \right)} \right]^t} } \right)$$

The prediction accuracy between two different cell types was calculated using 10-fold cross validations, which means that we had 10 training and 10 test sets. Then, we built the classifier *f* (*X*_*t*_) and calculated the prediction accuracy using the test set. This procedure was repeated 10 times to obtain the prediction value between two groups of data. The prediction value between two cell lines represents the accuracy of this machine learning algorithm to distinguish different cell types.

## Supplementary information

MICRONANO-00922-SI
