# Supplementary material for: Scalable nanolaminated SERS multiwell cell culture assay
Source: Microsyst Nanoeng. 2020 Jun 1;6:47. doi: 10.1038/s41378-020-0145-3 (PMC8433130; doi:10.1038/s41378-020-0145-3)
Supplement: Supplementary file 1 — MICRONANO-00922-SI [file 41378_2020_145_MOESM1_ESM.docx]

**Supplementary Information**

X. Ren, W. Nam, P. Ghassemi, J.S. Strobl, I. Kim, W. Zhou, M. Agah, *Scalable Nanolaminated SERS Multi-Well Cell Culture Assay*

1. Raman spectrum


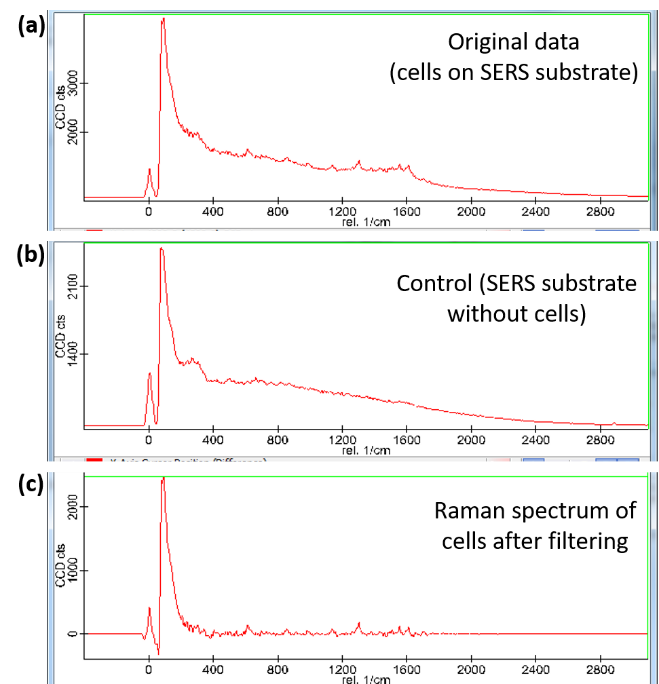


Figure S1. The Raman spectrum collection of cells: (a) measured data of cells on nanolaminated SERS substrate; (b) the Raman spectrum of the control group of SERS substrate without cells; (c) the Raman spectrum of cells by filtering the control group from the original data. The remaining spectrum is effective information of Raman spectrum of cells and the Rayleigh peak. The data we selected between 400~1800 cm^-1^ are Raman spectrum of cells.

2. SERS substrate fabrication

The entire fabrication process is schematically illustrated in Figure 1. UV-curable polyurethane (PU) (NOA83H, Norland Product Inc., USA) was used to fabricate a periodic nanopillar array (NPA) (diameter, d = 120 nm; period, a = 400 nm; height, h = 150 nm) by replica molding (soft-lithography). PU was squeezed onto a flexible and optically transparent polyester (PE) film whose thickness is 100 µm and then molded using a PDMS stamp. The sample was cured by UV for 10 min and the PDMS stamp was then peeled off (Figure 2 step ①). An additional heat-curing process was performed in a convection oven at 80°C overnight. Next, we deposited alternating layers of Au and SiO_2_ by electron-beam deposition (PVD250, Kurt J. Lesker Company, USA) on NPA (Figure 2 step ②). The nominal thicknesses of the four Au layers and the three SiO_2_ layers are 30 nm and 6, 8, and 12 nm from the bottom to top, respectively. In order to prevent collapse of multilayered nanostructures during the following wet etching process, we deposited 1 nm of Cr as an adhesion layer between NPA and the first layer of Au whereas 0.7 nm of Ti adhesion layer was deposited between every metal and insulator layer to be etched together with SiO_2_. 10:1 buffered oxide etchant (BOE) solution (Transene INC, USA) was used to etch SiO_2_ layers for 30 seconds (Figure 2 step ③). This process is to activate latent hot spots buried in the nanogaps, which is physically unapproachable for molecules before etching. 30 seconds of etching was optimized to achieve physical accessibility for molecules while maintaining the robustness of vertically stacked multilayers [^32^](#_ENREF_32). More than 30 seconds etching would induce collapse of nanoantennas and accordingly cause degradation of SERS performance. In this work, we call this novel nanostructure as multilayered MIM nanolaminated SERS substrate. The described fabrication method offers several advantages compared to other techniques for conventional SERS substrates: a facile, time-saving, and cost-effective method with the capability of incorporation with other modalities over large areas, which can be fabricated on different substrates. The photo image (a) in Figure 1 highlights representative sample size fabricated on a flexible and optically transparent substrate.
